# Use of the pro-seal laryngeal mask airway facilitates percutaneous dilatational tracheostomy in an intensive care unit

**DOI:** 10.4103/0972-5229.76082

**Published:** 2010

**Authors:** Suman Sarkar, P Shashi, Anil Kumar Paswan, R.P. Anupam, S. Suman, Surya Kumar Dube

**Affiliations:** **From:** Department of Anesthesiology, Institute of Post Graduation Medical Education and Research (I.P.G.M.E.R), Kolkata, West Bengal, India; 1 Department of Anesthesiology, Institute of Medical Sciences, Banaras Hindu University, Varanasi, Uttar Pradesh, India; 2 Department of Anesthesiology, Maulana Azad Medical College and Associated Lok Nayak and GB Panth Hospitals, New Delhi-110 002, India; 3 Department of Anesthesiology, All India Institute of Medical Sciences (AIIMS), New Delhi, India

**Keywords:** Laryngeal mask airway, percutaneous tracheostomy, pro-seal laryngeal mask airway

## Abstract

**Purpose:**

To study the feasibility of using the pro-seal laryngeal mask airway (LMA) for airway maintenance during bronchoscopic-guided percutaneous tracheostomy.

**Materials and Methods:**

Observational study of 60 patients in a 16-bed intensive care unit. The patient’s tracheal tube was exchanged for a pro-seal LMA before undertaking percutaneous tracheostomy.

**Results:**

Inspiratory pressure and tidal volumes achieved during the procedure were recorded. The median peak inspiratory pressure was 25 (standard deviation 4.4) cm H2O. There was no loss of tidal volume in 30 patients, a loss of less than 100 mL/breath in 27, and loss of more than 100 mL in 3 patients. A pro-seal LMA successfully maintained the airway and allowed adequate ventilation during per-cutaneous tracheostomy in all 60 patients. In all patients, fiber optic bronchoscopy through the pro-seal LMA provided a clear view of the cords and trachea and there was no laryngeal or tracheal soiling at any stage of the procedure.

**Conclusion:**

The pro-seal LMA provides a reliable airway and allows effective ventilation during percutaneous tracheostomy. The passage of a fiberscope through the pro-seal LMA and glottis is easy and provides a clear view of the upper trachea.

## Introduction

Using percutaneous dilatational tracheostomy the patient’s airway is usually maintained with an endo-tracheal tube, which is pulled back until the cuff is lying across the vocal cords.[1,2] In this position, the tracheal tube is unstable and may become dislodged with loss of the airway and risk of aspiration. Most commonly, fiber optic bronchoscopy is undertaken during the procedure to aid the correct placement of the guidewire.[3] An assistant is generally required to stabilize the tracheal tube while fiberoptic examination is performed and, as the glottis is not seen, it can be very difficult to determine the level at which the cannula enters the trachea. The tip of the tracheal tube may still lie low enough within the trachea to risk cuff puncture or tube transfixion by the needle or by the guide wire.[4] Use of the classic laryngeal mask airway (LMA)[5–7] or the intubating LMA[8] during percutaneous dilatational tracheostomy (PDT) circumvents many of these problems. However, patients requiring PDT often have poorly compliant lungs requiring high inflation pressures. Gas leak from between the LMA and glottis increases as inspiratory airway pressure rises, particularly above 20 cm H_2_O.[9] This leads to a risk of hypoventilation in patients with poorly compliant lungs.

**Figure 1:**
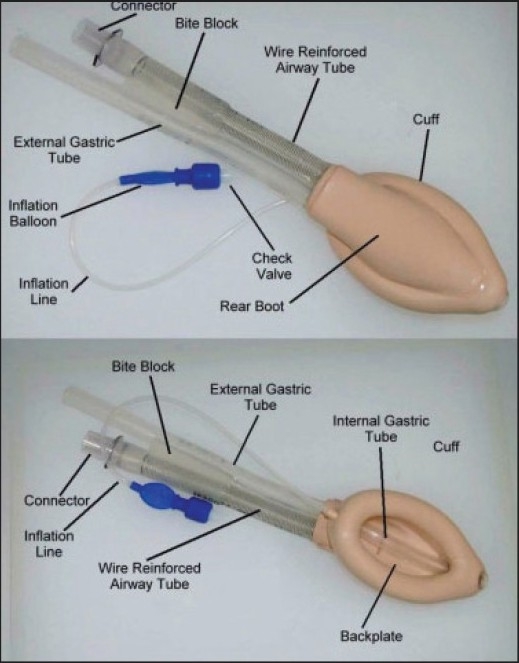
Pro-seal laryngeal mask airway

Pro-seal LMA has a drain tube lateral to the airway tube and ending at the mask tip[10] [Figure 1]. The second tube aids the correct placement of the mask; if the mask is placed correctly, with its tip against the upper esophageal sphincter, no air leak is heard. This tube allows the passage of a gastric tube and will vent air or fluid from the upper esophagus and stomach. The pro-seal LMA provides a better laryngeal seal than the classic LMA because of a deeper mask bowl and extension of the cuff over the posterior aspect of the bowl.[11] The aim of this observational study was to evaluate the efficacy of the pro-seal LMA during PDT under bronchoscopic guidance.

## Materials and Methods

### 

#### Patients

We prospectively studied 60 consecutive patients in the intensive care unit who required elective tracheostomy. The lungs of all patients were ventilated with a *Puritan-Bennett ventilator* using a positive airway pressure mode. We excluded those patients who needed inspiratory pressure more than 40 mmHg due to very bad lung pathology (pulmonary edema, collapse, ARDS, etc)

#### Technique

Before PDT, enteral feeding was withheld for 6 h in all patients and all of them were pre-medicated with metoclopramide i.v. as a prokinetic agent. All patients were sedated with propofol 100–200 μg/kg/min and fentanyl 0.5--1 μg/kg/h. Paralysis was achieved with vecuronium 0.1 mg/kg. The inspired oxygen concentration was increased to 100%. The pharynx was suctioned with a soft suction catheter. The nasogastric tube was aspirated and left *in situ* throughout the procedure. The patient was positioned with the neck extended and a pillow placed under the shoulders. A direct laryngoscopy done in all patients to assess the airways, after satisfactory findings, the tracheal tube was removed, a pro-seal LMA inserted (size 3 for females and size 4 for males), and the cuff inflated. Correct positioning of the pro-seal LMA was confirmed by the achievement of an adequate expired tidal volume with minimal leak from the drain tube and appearance of capnograph.

The patient’s neck was cleaned and draped. The cricoid cartilage was identified and the skin over the space between the first and second tracheal rings was infiltrated with 2% lignocaine with adrenaline. A fiberoptic bronchoscope was passed through a swivel connector on the pro-seal LMA. The tip of the bronchoscope was positioned just below the glottis and correct position of the tracheal puncture was confirmed in relation to both the midline and the level. A Cook ‘Blue Rhino’ PDT kit was used in all cases. The airway pressure and tidal volumes were recorded during insertion of the dilator and the presence of any gas leak was noted.

## Results

The pro-seal LMA successfully maintained the airway and allowed adequate ventilation during percutaneous tracheostomy in all 60 patients. In seven patients more than one attempt was required to insert the device. In five male patients the size 4 pro-seal LMA was changed to a size 5 and in three female patients the size 3 pro-seal LMA was changed to a size 4 to provide a better seal with the larynx. The median peak inspiratory pressure was 25 cm H_2_O (SD 4.4 cm H_2_O) and the median expired tidal volume was 400 mL (SD 91 mL). We have used Stela puda hozo *et al*, theory to detect SD from median.[12] There was no loss of tidal volume in 30 patients, minimal loss (less than 100 mL/breath) in 27, and moderate loss (200 mL) in 3. The lowest SpO_2_ recorded during the procedure was 88% (due some difficulty to insert PLMA and it took more than one attempt). The median lowest recorded SpO_2_ was 94%. Gastric fluid appeared in the drain tube during the procedure in six patients. In three of these cases this fluid was approximately 40 mL of enteral feeding solution (despite aspiration of the stomach before the procedure), and in the other it appeared to be bile. In these cases, the fluid was vented effectively up the drain tube. In all patients bronchoscopy through the pro-seal LMA provided a clear view of the cords and trachea and there was no laryngeal or tracheal soiling at any stage of the procedure.

## Discussion

Use of any form of LMA during PDT has the potential to overcome many of the problems associated with the procedure.[5–8] In particular, the airway device is in a stable position and lies outside the larynx minimizing the risks of dislodgement, glottic damage and tube transfixion.[4] Fiber optic Bronchoscopic examination may be performed from the laryngeal inlet allowing accurate identification of the laryngeal rings and avoiding risk of damage to the bronchoscope. In comparison with the classic LMA, the absence of aperture bars on the pro-seal LMA improves bronchoscopic access to the glottis during the procedure. In the presence of poor lung compliance and high airway pressures, the pro-seal LMA will provide more effective ventilation than the classic LMA[10,11] and it allows adequate ventilation in all 60 patients despite inspiratory airway pressures of up to 40 cm H_2_O.

Provisional work in cadavers, supported by some case reports, suggests that the drain tube of the pro-seal LMA will allow regurgitated matter to bypass the larynx.[13–15]

This was witnessed in four of our patients and, in all patients, laryngeal/tracheal soiling was absent. The potential for airway loss during changeover of the airway device is a theoretical limitation of the technique. However, accidental extubation during PDT may also cause loss of the airway as well as the risk of aspiration.

## Conclusion

Our experience suggests that the pro-seal LMA can be used to safely maintain the airway during PDT. Use of the pro-seal LMA during bronchoscopic-guided PDT facilitates a good view of the upper trachea and avoids the possibility of accidental extubation or puncture of the tracheal tube cuff.
